# Low-temperature redetermination of *trans*-cyclo­hexane-1,2-dicarboxylic acid

**DOI:** 10.1107/S1600536808012592

**Published:** 2008-05-03

**Authors:** Mohd. Razali Rizal, Seik Weng Ng

**Affiliations:** a Department of Chemistry, University of Malaya, 50603 Kuala Lumpur, Malaysia

## Abstract

The mol­ecule of the title compound, C_8_H_12_O_4_, lies on a twofold rotation axis that passes through the mid-points of two opposite C—C bonds of the ring. Carboxyl groups of adjacent mol­ecules are linked by pairs of hydrogen bonds around a centre of inversion; this inter­action gives rise to a chain that runs along [101].

## Related literature

Studies on the metal derivatives of *trans*-1,2-cyclo­hexa­ne­dicarboxylic acid refer to the room-temperature structure of Benedetti *et al.* (1969 ▶). The absence of a preferred orientation (either axial or equatorial) of the carboxyl groups in cyclo­hexa­nedicarboxylic acids is discussed in the case of 1,3-cyclo­hexanedicarboxylic acid by van Koningsveld (1984 ▶). For the crystal structure of 1,4-cyclo­hexa­nedicarboxylic acid, see: Luger *et al.* (1972 ▶).
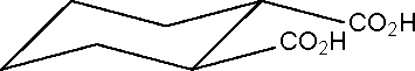


## Experimental

### 

#### Crystal data

C_8_H_12_O_4_
*M* *_r_* = 172.18
Monoclinic, 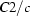

*a* = 5.585 (1) Å
*b* = 13.840 (3) Å
*c* = 10.035 (2) Å
β = 96.114 (3)°
*V* = 771.3 (3) Å^3^
*Z* = 4
Mo *K*α radiation
μ = 0.12 mm^−1^
*T* = 100 (2) K
0.38 × 0.06 × 0.04 mm

#### Data collection

Bruker SMART APEX diffractometer
Absorption correction: none
2320 measured reflections
883 independent reflections
715 reflections with *I* > 2σ(*I*)
*R*_int_ = 0.035

#### Refinement

*R*[*F* ^2^ > 2σ(*F* ^2^)] = 0.042
*wR*(*F* ^2^) = 0.115
*S* = 1.07
883 reflections
59 parameters
1 restraint
H atoms treated by a mixture of independent and constrained refinement
Δρ_max_ = 0.31 e Å^−3^
Δρ_min_ = −0.28 e Å^−3^

### 

Data collection: *APEX2* (Bruker, 2007 ▶); cell refinement: *SAINT* (Bruker, 2007 ▶); data reduction: *SAINT*; program(s) used to solve structure: *SHELXS97* (Sheldrick, 2008 ▶); program(s) used to refine structure: *SHELXL97* (Sheldrick, 2008 ▶); molecular graphics: *X-SEED* (Barbour, 2001 ▶); software used to prepare material for publication: *publCIF* (Westrip, 2008 ▶).

## Supplementary Material

Crystal structure: contains datablocks global, I. DOI: 10.1107/S1600536808012592/cv2404sup1.cif

Structure factors: contains datablocks I. DOI: 10.1107/S1600536808012592/cv2404Isup2.hkl

Additional supplementary materials:  crystallographic information; 3D view; checkCIF report

## Figures and Tables

**Table 1:** Hydrogen-bond geometry (Å, °)

*D*—H⋯*A*	*D*—H	H⋯*A*	*D*⋯*A*	*D*—H⋯*A*
O1—H1*o*⋯O2^i^	0.85 (1)	1.81 (1)	2.662 (2)	178 (2)

Symmetry code: (i) 
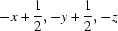
.

**Fig. 1.**
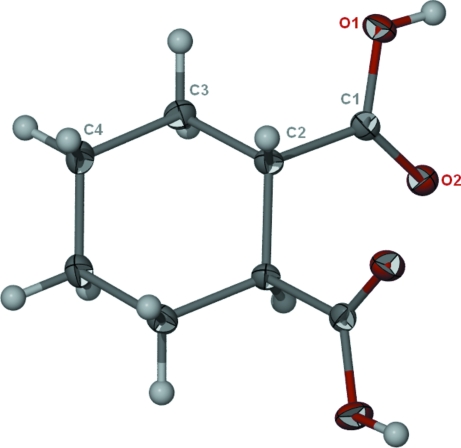
The molecular structure of the title compound with atomic numbering and 70% probability displacement ellipsoids. Hydrogen atoms are drawn as spheres of arbitrary radiius. The unlabeled atoms are related to the labeled ones by 1 - x, y, 1/2 - z.

**Fig. 2.**
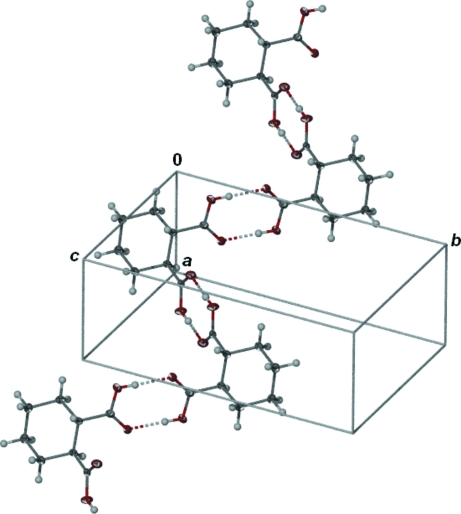
A portion of the crystal packing showing the hydrogen-bonded (dashed lines) chain.

## supplementary crystallographic information

### Comment

Crystallographic studies of the metal derivatives of *trans*-1,2-cyclohexanedicarboxylic acid occasionally refer to the room-temperature crystal structure of the dicarboxylic acid, which was reported in 1969. The report (Benedetti *et al.*, 1969) contains typographical errors that have since been corrected in the Cambridge Structural Database (Version 5.29, Nov. 2007). The reported monoclinic cell dimensions can be transformed to 5.65 (1), *b* 13.34 (3), *c* 10.22 (3) Å; β 97.2 (2)°.

Whereas the low-temperature unit cell has a slightly larger volume compared with the room-temperature cell, the low-temperature cell has a much longer *b*-axis [13.840 (3) Å]. The bond distances and angles of room-temperature structure are normal; those of the present study are not significantly different despite the longer axis. Possibly, the expansion of this axis is a genuine observation. Moreover, the present study is able to establish the hydrogen bonding scheme of the compound (Scheme I, Fig. 1). Adjacent molecules are linked by a linear O–H···O hydrogen bond [2.662 (2) Å] into a chain (Fig. 2).

The crystal structures of 1,3- and 1,4-cyclohexanedicarboxylic acids have already been reported (van Koningsveld, 1984; Luger *et al.*, 1972).

### Experimental

The commercially available acid was recrystallized from ethanol.

### Refinement

Carbon-bound H-atoms were placed in calculated positions (C—H 0.99 to 1.00 Å) and were included in the refinement in the riding model approximation, with *U*(H) set to 1.2 *U*(C). The acid H-atom was located in a difference Fourier map, and was isotropically refined with a distance restraint of O–H 0.85 (1) Å.

### Crystal data

**Table:**

C_8_H_12_O_4_	*F*_000_ = 368
*M**_r_* = 172.18	*D*_x_ = 1.483 Mg m^−^^3^
Monoclinic, *C*2/*c*	Mo *K*α radiation λ = 0.71073 Å
Hall symbol: -C 2yc	Cell parameters from 739 reflections
*a* = 5.585 (1) Å	θ = 3.6–28.2º
*b* = 13.840 (3) Å	µ = 0.12 mm^−^^1^
*c* = 10.035 (2) Å	*T* = 100 (2) K
β = 96.114 (3)º	Strip, colourless
*V* = 771.3 (3) Å^3^	0.38 × 0.06 × 0.04 mm
*Z* = 4	

### Data collection

**Table:**

Bruker SMART APEX diffractometer	715 reflections with *I* > 2σ(*I*)
Radiation source: fine-focus sealed tube	*R*_int_ = 0.035
Monochromator: graphite	θ_max_ = 27.5º
*T* = 100(2) K	θ_min_ = 2.9º
ω scans	*h* = −7→7
Absorption correction: None	*k* = −17→17
2320 measured reflections	*l* = −13→8
883 independent reflections	

### Refinement

**Table:**

Refinement on *F*^2^	Secondary atom site location: difference Fourier map
Least-squares matrix: full	Hydrogen site location: inferred from neighbouring sites
*R*[*F*^2^ > 2σ(*F*^2^)] = 0.042	H atoms treated by a mixture of independent and constrained refinement
*wR*(*F*^2^) = 0.115	*w* = 1/[σ^2^(*F*_o_^2^) + (0.0635*P*)^2^ + 0.2009*P*] where *P* = (*F*_o_^2^ + 2*F*_c_^2^)/3
*S* = 1.07	(Δ/σ)_max_ = 0.001
883 reflections	Δρ_max_ = 0.31 e Å^−^^3^
59 parameters	Δρ_min_ = −0.28 e Å^−^^3^
1 restraint	Extinction correction: none
Primary atom site location: structure-invariant direct methods	

### Fractional atomic coordinates and isotropic or equivalent isotropic displacement parameters (Å^2^)

**Table:**

	*x*	*y*	*z*	*U*_iso_*/*U*_eq_	
O1	0.0840 (2)	0.1596 (1)	0.0902 (1)	0.0173 (3)	
O2	0.4725 (2)	0.1968 (1)	0.0869 (1)	0.0166 (3)	
C1	0.3157 (3)	0.1471 (1)	0.1279 (2)	0.0122 (3)	
C2	0.3668 (2)	0.0626 (1)	0.2220 (2)	0.0122 (4)	
C3	0.2893 (3)	−0.0314 (1)	0.1475 (2)	0.0139 (4)	
C4	0.3647 (3)	−0.1208 (1)	0.2303 (2)	0.0159 (4)	
H1o	0.068 (4)	0.206 (1)	0.035 (2)	0.036 (6)*	
H2	0.2676	0.0704	0.2986	0.015*	
H3a	0.1121	−0.0315	0.1261	0.017*	
H3b	0.3626	−0.0339	0.0620	0.017*	
H4a	0.2799	−0.1218	0.3120	0.019*	
H4b	0.3184	−0.1796	0.1776	0.019*	

### Atomic displacement parameters (Å^2^)

**Table:**

	*U*^11^	*U*^22^	*U*^33^	*U*^12^	*U*^13^	*U*^23^
O1	0.0124 (6)	0.0179 (6)	0.0205 (7)	0.0008 (4)	−0.0027 (5)	0.0074 (5)
O2	0.0149 (6)	0.0153 (6)	0.0186 (6)	−0.0017 (4)	−0.0028 (4)	0.0048 (4)
C1	0.0140 (7)	0.0112 (7)	0.0107 (8)	0.0012 (5)	−0.0025 (6)	−0.0034 (6)
C2	0.0115 (7)	0.0118 (7)	0.0126 (8)	0.0003 (5)	−0.0025 (6)	−0.0004 (6)
C3	0.0138 (7)	0.0141 (7)	0.0134 (8)	−0.0013 (5)	−0.0012 (6)	−0.0014 (6)
C4	0.0170 (8)	0.0109 (7)	0.0190 (9)	−0.0011 (5)	−0.0012 (6)	0.0003 (6)

### Geometric parameters (Å, °)

**Table:**

O1—C1	1.321 (2)	O1—H1o	0.85 (1)
O2—C1	1.220 (2)	C2—H2	1.0000
C1—C2	1.511 (2)	C3—H3a	0.9900
C2—C2^i^	1.533 (3)	C3—H3b	0.9900
C2—C3	1.5397 (19)	C4—H4a	0.9900
C3—C4	1.523 (2)	C4—H4b	0.9900
C4—C4^i^	1.521 (3)		
			
O2—C1—O1	123.1 (1)	C3—C2—H2	108.3
O2—C1—C2	123.6 (1)	C4—C3—H3a	109.2
O1—C1—C2	113.3 (1)	C2—C3—H3a	109.2
C1—C2—C2^i^	109.9 (1)	C4—C3—H3b	109.2
C1—C2—C3	109.0 (1)	C2—C3—H3b	109.2
C2^i^—C2—C3	112.9 (1)	H3a—C3—H3b	107.9
C4—C3—C2	112.0 (1)	C4^i^—C4—H4a	109.5
C4^i^—C4—C3	110.5 (1)	C3—C4—H4a	109.5
C1—O1—H1o	109 (1)	C4^i^—C4—H4b	109.5
C1—C2—H2	108.3	C3—C4—H4b	109.5
C2^i^—C2—H2	108.3	H4a—C4—H4b	108.1
			
O2—C1—C2—C2^i^	11.2 (2)	C1—C2—C3—C4	172.7 (1)
O1—C1—C2—C2^i^	−171.2 (1)	C2^i^—C2—C3—C4	50.2 (2)
O2—C1—C2—C3	−113.0 (2)	C2—C3—C4—C4^i^	−56.6 (2)
O1—C1—C2—C3	64.6 (2)		

Symmetry codes: (i) −*x*+1, *y*, −*z*+1/2.

### Hydrogen-bond geometry (Å, °)

**Table:**

*D*—H···*A*	*D*—H	H···*A*	*D*···*A*	*D*—H···*A*
O1—H1o···O2^ii^	0.85 (1)	1.81 (1)	2.662 (2)	178 (2)

Symmetry codes: (ii) −*x*+1/2, −*y*+1/2, −*z*.
